# Prophylaxis of Diallyl Disulfide on Skin Carcinogenic Model *via* p21-dependent Nrf2 stabilization

**DOI:** 10.1038/srep35676

**Published:** 2016-10-19

**Authors:** Yunlong Shan, Zhonghong Wei, Li Tao, Siliang Wang, Feng Zhang, Cunsi Shen, Hongyan Wu, Zhaoguo Liu, Pingting Zhu, Aiyun Wang, Wenxing Chen, Yin Lu

**Affiliations:** 1 Jiangsu Key Laboratory for Pharmacology and Safety Evaluation of Chinese Materia Medica, School of Pharmacy, Nanjing University of Chinese Medicine, Nanjing 210023, China; 2 Jiangsu Collaborative Innovation Center of Traditional Chinese Medicine (TCM) Prevention and Treatment of Tumor, Nanjing University of Chinese Medicine, Nanjing 210023, China

## Abstract

Cancer prevention through intake of biologically active natural products appears to be an accessible way to reduce the risk of cancer. Diallyl disulfide (DADS), a major garlic derivative, has exhibited potential role in cancer therapy. The study is aimed to evaluate the prophylactic effect of DADS in chemically induced mouse skin carcinogenesis and investigate the molecular targets mediated by DADS. Two-stage chemically induced carcinogenesis model by cutaneous application of DMBA and subsequent TPA was established to study the prophylactic effect of DADS. As a result, we observed that DADS dose-dependently attenuated skin tumor incidence and multiplicity in the model mice, which was related to the up-regulation of a bunch of antioxidant enzymes activities and the nuclear accumulation of Nrf2. Furthermore, we developed skin carcinogenesis in Nrf2 knockout mice which could reverse the activity of DADS. Finally, we uncovered the underlying mechanism that DADS promoted the endogenous interaction between p21 and Nrf2, which was critical for impairing the Keap1-mediated degradation of Nrf2. Based on the results, we concluded that DADS was a promising cancer chemoprevention agent and suggested a garlic-rich diet might be beneficial to reduce the cancer risk in our daily life.

The challenges of conventional anticancer therapies include not only the frequent failure of clinical outcomes, but also the out-of-pocket expenditure for patients. Cancer chemoprevention is the use of substances to to slow down or eliminate the progression of intra epithelial neoplastic or precancerous lesions to tumor^1^, and these substances for cancer prevention should be nontoxic, economical, orally active and easily available^2^,^3^.

Of note, garlic as a popular and common edible food for flavoring in cooking, has been found an association with reduced risk of certain cancers^4^. DADS, a major organosulfur compound found in raw garlic and black garlic, has demonstrated a variety of pharmacological and biological activities, including anti-inflammatory^5^, antimicrobial^6^, and antitumor activities^7^. However, the prophylaxis and underlying mechanism of DADS on carcinogenesis are yet to be answered. To this end, the present study was aimed to utilize a two-stage chemically induced papillomagenesis in the back skin of mice, which involves topically use of 9,10-dimethylbenz[α]anthracene (DMBA), a tumor initiator that induces DNA damage, followed by 12-O-tetradecanoylphorbol-13-acetate (TPA), a phorbol ester that promotes tumor development^8^, for characterizing the potential chemopreventive effect of DADS.

The transcription factor NF-E2-related factor 2 (Nrf2) plays a vital role in maintaining cellular redox homeostasis, especially upon the exposure of cells to chemical or oxidative stress, through its ability to regulate the basal and inducible expression of a multitude of antioxidant proteins, detoxification enzymes and xenobiotic transporters^9^,^10^. However, this innate mechanism of skin cytoprotection is often diminished or become inadequate to alleviate cellular transformation induced by radiation or some chemical carcinogens^11^. Therefore, we focused on DADS-evoked antioxidant defense mechanism to combat the carcinogens, which might clue the molecular mechanisms that mediate the action of DADS.

Despite the immense efforts have suggested that diet derived phytochemicals are the most acceptable and promising agents for cancer prevention, there remains great demand for identifying the correct molecular targets and then successfully translate preclinical results into the clinical setting and further into the application of daily life. Herein, we utilized Nrf2^−/−^ mice to identify that DADS inhibited chemically induced mouse papillomagenesis through promoting the Nrf2 nuclear accumulation and elevating the expression of antioxidant enzymes. Moreover, we found the endogenous interaction of p21 and Nrf2 was enhanced after DADS treatment, which consequently protected Nrf2 from proteasomal degradation by Kelch-like ECH-associated protein 1 (Keap1). Thus, our data highlight the significance of DADS in cancer prevention and consumption of garlic as dietary supplement is healthy life style to reduce the cancer risk.

## Results

### DADS exhibits prophylactic effect on DMBA/TPA-induced mouse skin carcinogenesis

**Figure 1:**
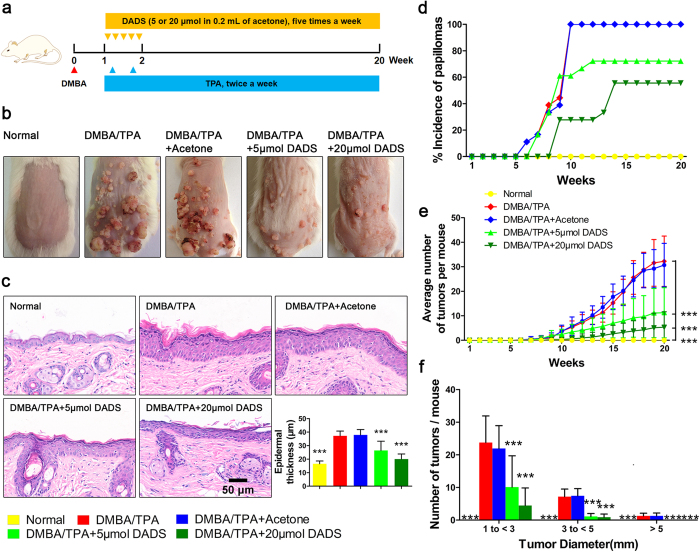
Chemopreventive effect of DADS on DMBA/TPA-promoted skin cancer *in vivo* and *in vitro*. (**a**) The workflow of animals study as described in Material and Methods. (**b**) Representative images of papillomagenesis in the indicated groups. (**c**) Representative images of epidermal proliferation and hyperplasia in the indicated groups. (Bottom Right) Quantitative analysis of H&E data (n = 9). (**d**) The incidence of papillomas in different treatment groups (n = 18). (**e**) The average numbers of papillomas per mouse in the indicated groups (n = 18). (**f**) The average numbers of papillomas per mouse in different tumor diameter groups (n = 18). The data are presented as the mean ± SD. ***p < 0.001 (versus DMBA/TPA).

To identify the chemopreventive effect of DADS, we first performed DMBA-initiated and TPA-promoted mouse skin carcinogenesis *in vivo*. The representative images of dorsal skin from all the indicated groups in Fig. 1b showed that DMBA/TPA successfully induced papillomagenesis, which could be greatly inhibited by DADS treatment in a concentration-dependent manner. Histological analysis further signified that DADS notably attenuated the increase in epidermal thickness (hyperplasia) induced by DMBA/TPA (Fig. 1c). Furthermore, compared with the DMBA/TPA-treated group, pretreatment with DADS remarkably reduced both the incidence (Fig. 1d) and multiplicity (Fig. 1e) of skin papilloma formation, and it substantially delayed the latency period from 5 to 8 (20 μmol) weeks. In addition, the inhibition of DADS on tumorigenesis was demonstrated by the papilloma size distribution (Fig. 1f). Supporting evidence showed that DADS suppressed TPA-induced JB6 P+ cells transformation *in vitro* (Supplementary Figure S1). Taken together, the animal study strongly suggested DADS efficiently prevented DMBA-initiated and TPA-promoted mouse skin carcinogenesis.

### DADS triggers the antioxidant response *in vivo* and *in vitro*

**Figure 2:**
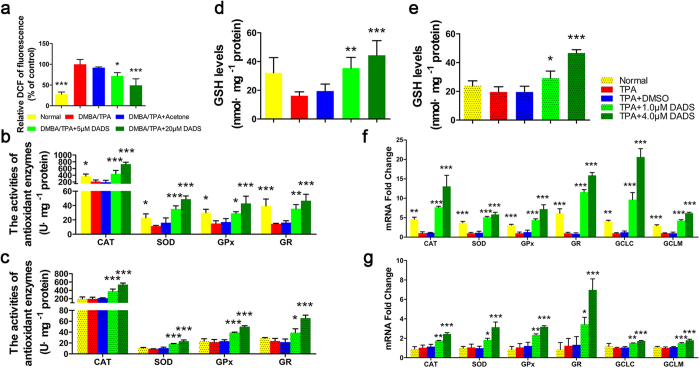
DADS increased the activities and mRNA levels of antioxidant enzymes. (**a**) The DCFH-DA staining was used to detect ROS production in the indicated groups (n=3). (DMBA/TPA as control) (**b**) The activities of CAT, SOD, GPx and GR in mouse skin of the indicated groups (n = 3). (**c**) JB6 P+ cells were pretreated with DADS at 4 μmol/L for 12 hours and then exposed to 20 ng/mL TPA for additional 2 hours. The activities of CAT, SOD, GPx and GR were induced by DADS *in vitro* (n = 3). (**d**) Effect of DADS on GSH levels in mouse skin from the indicated groups (n = 3). (**e**) The effect of DADS on GSH levels in DADS-pretreated JB6 P+ cells with TPA stimulation (n = 3). (**f**) Total mRNA was isolated and analyzed to determine the levels of CAT, SOD, GPx, GR, GCLC and GCLM expression using real-time qPCR after DADS treatment in mouse skin of the indicated groups (n = 3). (**g**) The mRNA levels of CAT, SOD, GPx, GR, GCLC and GCLM were detected by real-time qPCR in DADS-pretreated JB6 P+ cells with TPA stimulation (n = 3). GAPDH was used as the loading control. The data are presented as the mean ± SD. *p < 0.05, **p < 0.01 and ***p < 0.001 (versus DMBA/TPA or TPA).

The protective effects of DADS are thought to be associated with their antioxidant properties in various diseases^12^,^13^,^14^. Pre-treatment with DADS significantly inhibited the accumulation of reactive oxygen species (ROS) in a dose-dependent manner compared to the DMBA/TPA-treated group (Fig. 2a). The antioxidant enzyme system, including superoxide dismutase (SOD), heme oxygenase-1 (HO-1), glutathione peroxidase (GPx), catalase (CAT) and glutathione (GSH), is a key molecular target for chemopreventive agents to eliminate ROS^15^. Therefore, we further asked whether DADS could trigger cellular antioxidant response in the presence of DMBA/TPA. Expectedly, our results exhibited that DADS could rescue the antioxidant deficiency induced by carcinogen through increasing the activities of the antioxidant enzymes including CAT, SOD, GPx and GR (Fig. 2b,c) and the tissue and cellular levels of GSH, respectively (Fig. 2d,e). Moreover, the mRNA levels of antioxidant enzymes including CAT, SOD, GPx, GR, glutamate—cysteine ligase catalytic subunit (GCLC) and glutamate-cysteine ligase modifier subunit (GCLM) were significantly up-regulated after DADS treatment (Fig. 2f,g), suggesting a *de novo* synthesis of theses enzymes served as an important salvage pathway to escape the detrimental effects of DMBA/TPA and protect cells from malignant transformation. Altogether, our data indicated that activation of antioxidant system was responsible for the cancer chemoprevention of DADS.

### Nrf2 is required for DADS-induced cancer prevention in mouse skin

**Figure 3:**
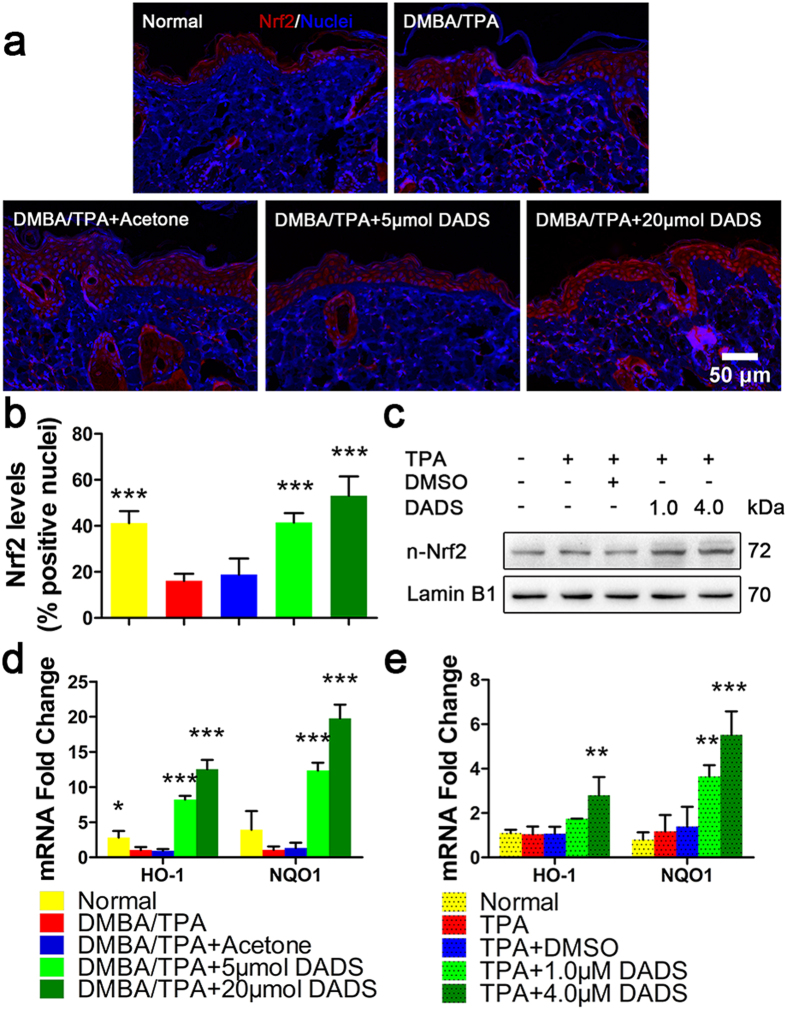
Nuclear accumulation and signaling activity of Nrf2 was induced by DADS *in vivo* and *in vitro*. (**a**) IHC analysis of Nrf2 expression in mouse epidermis in different treatment groups (blue, nuclei; red, Nrf2). (**b**) Quantitative analysis of IHC data (n = 9). (**c**) JB6 P+ cells were pretreated with DADS at 4 μM for 12 hours and then exposed to 20 ng/mL TPA for additional 2 hours, and the nuclear levels of Nrf2 and LaminB1 were measured by Western blotting. LaminB1 was used as the loading control. (**d**) Quantitative RT-PCR analysis of Nrf2 target genes in the mouse skin of the indicated groups. (e) mRNA levels of HO-1 and NQO1 were detected by real-time qPCR in JB6 P+ cells. GAPDH was used as the loading control. The data are presented as the mean ± SD. *p < 0.05, **p < 0.01 and ***p < 0.001 (versus DMBA/TPA or TPA).

In view of the critical role of Nrf2 in regulating redox imbalance^16^, we further examined whether the Nrf2 signaling was involved in the prophylactic effect of DADS. As results, DADS obviously induced nuclear accumulation of Nrf2 in the epidermis (Fig. 3a,b). Consistent with this finding, DADS also increased the nuclear localization of Nrf2 *in vitro*, as shown in Fig. 3c. Next, we found coordinated upregulation of HO-1 and NQO1 transcription, two Nrf2 specific target genes controlled by DADS (Fig. 3d,e). These findings suggested DADS-mediated activation of antioxidant enzymes was associated with increased functional Nrf2.

**Figure 4:**
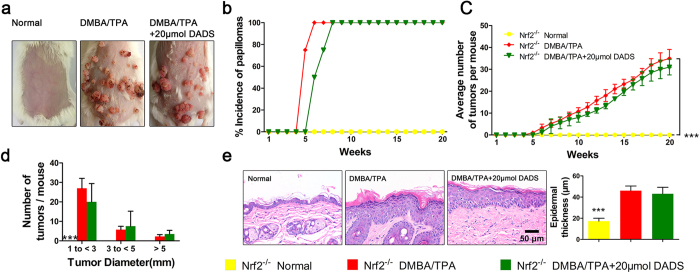
Nrf2 was required for DADS-induced cancer prevention in mouse skin *in vivo*. (**a**) Animals were treated as described in Fig. 1. Physical appearance of representatives of the indicated groups. (**b**) Tumor morphology of dorsal skin in different treatment groups (n = 4). (**c**) Average numbers of papillomas per mouse from the indicated groups (n = 4). (**d**) Average numbers of papillomas per mouse in different tumor diameter groups (n = 4). (**e**) H&E staining mouse skin to represent the whole skin structure. (Right) Quantitative analysis of H&E data (n = 12). The data are presented as the mean ± SD. ***p < 0.001 (versus DMBA/TPA).

To further verify whether Nrf2 was a prerequisite target for the chemoprevention of DADS, we further established DMBA/TPA-induced skin carcinogenesis in Nrf2 global deletion mice. Interestingly, we failed to observe the chemopreventive effect of DADS in absence of Nrf2. The skin morphology, size, and number of papilloma could not be attenuated by DADS in Nrf2^−/−^ mice (Fig. 4a–d). Moreover, there was no significant change of epidermal thickness in the DMBA/TPA-treated Nrf2^−/−^ group and DADS-treated Nrf2^−/−^ group (Fig. 4e). Similar to epidermal thickness, there was no any change in the levels of ROS, the mRNA levels of Nrf2 target enzymes and the levels of GSH (Supplementary Figure S2). Hence, these data confirmed that Nrf2 played a pivotal role in DADS-induced cancer prevention in mouse skin.

### DADS facilitates the interaction between p21 and Nrf2 that confers Nrf2 stabilization

**Figure 5:**
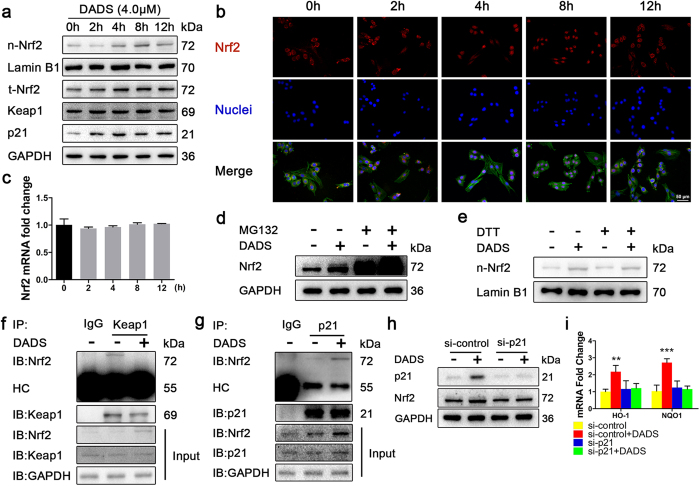
DADS facilitates the interaction between p21 and Nrf2 that confers Nrf2 stabilization. (**a**) Cells were treated with DADS (4 μM) and harvested at the indicated times, and analyzed to determine the nuclear Nrf2 levels and the expression of both total forms of Nrf2, Keap1 and p21 by Western blotting. (**b**) Treatment similar to (**a**), the immunofluorescence staining of Nrf2 was conducted as described in Materials and Methods (blue, nuclei; red, Nrf2, green, F-actin). (**c**) Total mRNA was isolated and analyzed to determine the levels of Nrf2 expression using real-time qPCR after DADS treatment. GAPDH was used as the control. The data are presented as the mean ± SD. (versus 0h) (**d**) Cells were treated with DADS in the presence or absence of MG132 (10 μM), a proteasome inhibitor, for 4 h. The level of Nrf2 was determined in whole cell lysates by Western blotting. GAPDH was used as the loading control. (**e**) Cells were incubated with DADS (4 μM) in the presence or absence of DTT (500 μM).The nuclear localization of Nrf2 were determined by Western blot analysis. (**f**) Cells were treated with DADS for 4 h. Co-IP was done as described in Materials and Methods, and the immunoprecipitates were immunoblotted using antibodies for Nrf2, Keap1 and GAPDH. (**g**) The immunoprecipitates were immunoblotted using antibodies for Nrf2, p21 and GAPDH. (**h**) HEK293T cells transfected with control siRNA or p21 siRNA were incubated with DADS for 8 h to determine the levels of p21 and Nrf2. (**i**) The mRNA levels of HO-1 and NQO1 were detected by real-time qPCR in HEK293T cells of the indicated groups. GAPDH was used as the loading control. The data are presented as the mean ± SD. **p < 0.01 and ***p < 0.001 (versus si-control).

We then examined the underlying molecular mechanism of Nrf2 translocation induced by DADS in JB6 P+ cells. As shown in Fig. 5a, the nuclear Nrf2 level was also elevated by DADS treatment in time-dependent. The nuclear accumulation of Nrf2 was further confirmed by immunofluorescence staining (Fig. 5b). Moreover, DADS failed to induce Nrf2 nuclear translocation through activation of upstream MAPK and Akt (Supplementary Figure S3). Similarly, Western blot analysis revealed that DADS treatment resulted in a significant increase in the total Nrf2 level (Fig. 5a). To understand how DADS-induced Nrf2 accumulation was executed, we then mainly focused on the post-translational regulation of Nrf2 which was evidenced by our observation that DADS did not alter the mRNA level of Nrf2 (Fig. 5c). Thus, we precluded that DADS-induced increase in total Nrf2 expression was not due to transcriptional modulation. Moreover, we introduced the MG132, a proteasome inhibitor to check whether the DADS-mediated Nrf2 stabilization was the result of blockage of its proteasomal degradation, and we found DADS increased Nrf2 protein levels partially through a proteasome-dependent pathway (Fig. 5d).

Keap1 anchors the Nrf2 transcription factor within the cytoplasm targeting it for ubiquitination and proteasomal degradation^17^, while cyclin-dependent kinase inhibitor p21, which was reported to positively influence Nrf2 signaling by binding to Nrf2 and thereby preventing it from binding to Keap1 and thwarting Nrf2 ubiquitination and degradation^18^. We next examined the role of p21 mediating the Nrf2 stability regulation and proposed p21-dependent Nrf2 induction by DADS. Supporting the notion, p21 might participate in the Nrf2 regulation as we observed that DADS elicited a time-dependent induction of p21 which was almost synchronized with the induction of Nrf2 (Fig. 5a). And DADS could upregulate the mRNA level of p21 in cells (Supplementary Figure S4). However, we could not observe the change of Keap1 expression level (Fig. 5a). The effects of DADS on the nuclear translocation of Nrf2 were not abrogated in cells pre-treated with the thiol reducing agent DTT (Fig. 5e). We next investigated the status of the p21 and Nrf2 complex in the presence of DADS. As we found in co-IP study, DADS induced the dissociation of Keap1 with Nrf2, accompanied by increased interaction of p21 with Nrf2, which implied that DADS-mediated interaction of p21 with Nrf2 rescued Nrf2 from Keap1-mediated ubiquitination and degradation (Fig. 5f,g). We then examined whether p21 indeed mediate the DADS-induced expression of Nrf2. HEK293T cells were transiently transfected with control siRNA or p21 siRNA, and incubated with DADS. As compared to cells transfected with scrambled siRNA, DADS-induced expression of p21 and Nrf2 was abrogated in cells transfected with p21 siRNA (Fig. 5h). Finally, we found that DADS could not upregulate the mRNA levels of HO-1 and NQO1 in the cells transfected with p21 siRNA (Fig. 5i). Collectively, these data suggested that recruitment of p21 to release Nrf2 from Keap1 was a primary mechanism of DADS-mediated Nrf2 stabilization.

## Discussion

Carcinogenesis is generally recognized as a multistep process that consists of initiation, promotion and progression^19^. Chemoprevention appears to be one of the most feasible approaches to inhibit, reverse or retard carcinogenesis. Cancer preventive measures to reduce the cancer risk are far more easier than treatment itself^20^. However, research efforts on drugs to prolong the life expectancy of late-stage cancer patients are driven by profit now. It can be approved in a few months which prolonging the life expectancy of late-stage cancer patients on drugs. But it takes more time to determine its effectiveness that focus on prevention.

Although a number of chemopreventive phytochemicals can block or reverse the premalignant stage of multistep carcinogenesis^21^, most preclinical studies using natural compounds were conducted using fully transformed cancer cell lines due to the lack of pre-malignant cell lines^1^. In the present study, we confirmed the potency in preventing carcinogenesis of DADS in pre-malignant JB6 P+ cell lines and a chemically induced mouse skin tumor model.

For the past decades, investigators have focused on DADS because it has been shown to decrease the formation of carcinogen-induced cancers and to inhibit the proliferation of various types of cancer cells^7^,^22^,^23^,^24^,^25^,^26^. Furthermore, DMBA-initiated and TPA-promoted mouse skin tumorigenesis is pivotal for the investigation of cancer prevention. Although studies showed that DADS suppressed the DMBA/TPA skin carcinogenesis in mice^27^, to our knowledge, the mechanisms underlying the prophylaxis of DADS have not yet been elucidated. In the present study, we used Nrf2^−/−^ mice to examine the hypothesis that DADS might exert its protective effects against skin tumors by triggering the antioxidant response. Our findings revealed that the incidence and the multiplicity of papillomas were lower than Dwivedi’s study on account of DADS treatment five times a week. The significant reduction of papillomas in chemically induced skin tumor suggested DADS as a potential cancer chemopreventive agent. In addition, we further confirmed the detailed molecular mechanisms that DADS exerted antitumor-promoting effect *in vivo* and *in vitro*. After clarifying the direct function of DADS on skin cancer cell, we will carry out experiments to explore the cancer chemoprevention of DADS on other organ, including lung, liver, pancreas and colon.

Previous studies demonstrated that diminished activity of antioxidant enzyme is associated with skin carcinogenesis^28^,^29^,^30^. Besides, emerging evidence has shown cytoprotective enzymes regulated through the evocation of antioxidant-responsive elements (ARE) mediated by activating nuclear transcription factor Nrf2 is one of several promising strategies to prevent cancer^15^,^16^. In the present study, we observed a reduction in Nrf2 nuclear translocation in cells treated with DMBA/TPA. This could be explained by activation of Nrf2 inhibitory factors^31^ or mutation of Nrf2 residues^32^ caused by DMBA or TPA, leading to increased nuclear export and proteasomal degradation of Nrf2 as well as inhibited nuclear translocation. The exact mechanisms await to be further defined. While our observation in line with Lee^33^,^34^, which showed that the increasing activities of antioxidant enzymes by DADS orally, at least in part, through inducing Nrf2 nuclear location in hepatic damage in rats, but nonetheless how DADS elevating activities of enzymes is linked to transcriptional regulation of Nrf2 is unclear at present. By utilizing transgenic mice, we were able to show that Nrf2 is essential for DADS attenuated tumorgenesis in mouse skin. Strikingly, DADS treatment tended to inhibit papillomagenesis in Nrf2^−/−^ mice, but this decrease was not statistically significant. This phenomenon may account for induction of cell-cycle arrest and apoptosis properties of DADS^35^.

The Nrf2 signaling pathway is negatively controlled by Keap1, and Nrf2 is sequestered in the cytoplasm under non-stimulated conditions by Keap1. Meanwhile, Keap1 constantly targets Nrf2 leading to ubiquitin-dependent degradation and thereby maintains low levels of Nrf2^36^,^37^,^38^. Pretreatment with DADS also induced accumulation of Nrf2 protein level, and our results were in accordance with previous observations^39^. Nevertheless, there was no change in mRNA level of Nrf2 by DADS in the present study. Moreover, MG132 dependency wad further solidified as the decreasing degradation effect of Nrf2 was found in cell treatment with DADS. Previously, Kim *et al*. reported that DATS might directly interact with the Cys288 residue of Keap1^40^. On the contrary, DADS still increased the nuclear translocation of Nrf2 in the presence of DTT in the present study. This difference between DADS and DATS was presumably due to different conformation and number of sulfur atoms in their chemical structure. Previous studies conducted by Zhang and his colleagues showed that p21 upregulated the Nrf2 by directly interfering with Keap1 recognition of Nrf2^18^. In addition, a large body of literature shows DADS is able to increase p21 expression in cancer cells^4^,^41^,^42^. As an initial approach to test the possibility of DADS-mediated Nrf2 activation through induction of p21, we utilized Co-IP capable of detecting the increased Nrf2 direct interaction with p21. Besides, p21 is induced in both a p53-dependent and -independent manner at the transcriptional and posttranscriptional levels in response to oxidative stress^43^. The present study indicated that the upregulation of p21 mRNA by DADS was attributed to the activation of p53. Supplementary Information Figure S5 showed that DADS-induced accumulation of Nrf2 was reversed by deletion of p53 protein in NCI-H1299 cells. Thus, it is worthwhile to further investigate the effects of DADS on the activation of p53 in mouse skin cells. While using specific agents targeted one of pathways may not be effective or durable, DADS may be a prospective agent for multi-targeted prevention and treatment against human disease.

**Figure 6:**
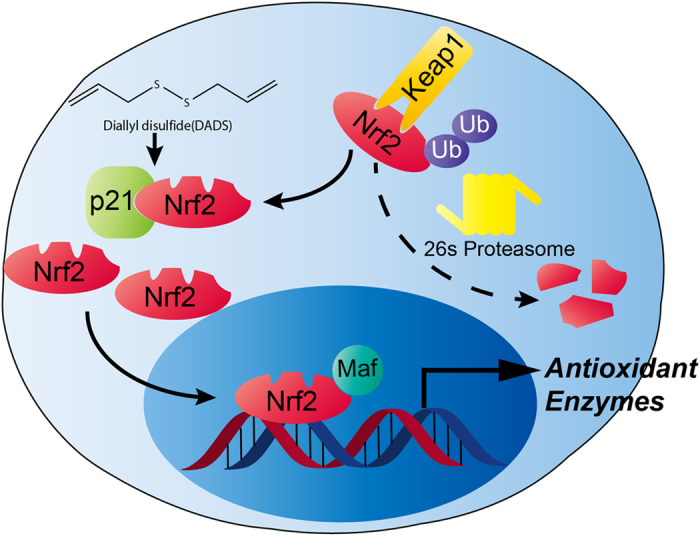
Mechanism for the role of Nrf2 signaling regulated by DADS in skin carcinogenesis.

In conclusion, our discovery are briefly summarized as follows (Fig. 6): topical application of DADS inhibits TPA-induced tumor promotion by means of promoting Nrf2 nuclear localization in mouse skin, which seemed to be mediated through suppressing degradation of Nrf2 *via* up-regulation of p21 protein level. The present study will provide insight into the future application of naturally occurring phytochemicals- DADS in the clinical setting.

## Methods

### Chemicals and reagents

DADS was purchased from LKT Laboratories. The 8 mM stock of DADS solution was prepared in 100% DMSO and cells treated with equal amount of DMSO served as vehicle. TPA was obtained from Cayman Chemical company. DMBA was purchased from Sigma Aldrich. Fetal Bovine Serum (FBS), minimum essential medium (MEM), and trypsin-EDTA solution were purchased from Gibco Laboratories. The reactive oxygen species (ROS) assay kit, CAT assay kit, GPx assay kit, GSH assay kit, SOD assay kit and GR assay kit were procured from Beyotime, China. siRNA for p21 and non-targeting control were purchased from Santa Cruz. DL-Dithiothreitol solution was obtained from SIGMA-ALDRICH.

### Cell line and cell culture

The mouse epidermal cell line, JB6 P+ (JB6 Cl 41-5a), from American Type Culture Collection (ATCC) were maintained in MEM containing 10% FBS in a humidified 5% CO_2_ atmosphere at 37 °C. The JB6 P+ epidermal cells are derived from mouse skin and are regarded as an appropriate cell model for studying the chemopreventive effect and underling mechanisms of DADS *in vitro*. HEK293T cell line from ATCC were maintained in DMEM containing 10% FBS in a humidified 5% CO_2_ atmosphere at 37 °C.

### Establishment of carcinogenesis model induced by DMBA/TPA

Female Institute of Cancer Research (ICR) mice (6–7 wk) were supplied from National Rodent Laboratory Animal Resources Shanghai Branch and housed in climate-controlled quarters with a 12-h light/12-h dark cycle. All experimental procedures were carried out in accordance with the Guide for the Care and Use of Laboratory Animals, and before the animal experiments were carried out, the procedures were approved by the Research Ethical Committee of Nanjing University of Chinese Medicine.

ICR mice were randomly divided into five groups, 18 animals per group. The workflow of the *in vivo* study was depicted in Fig.1a. Specifically, the groups receiving DMBA/TPA, DMBA/TPA/Acetone or DMBA/TPA/DADS were subjected to DMBA (60 μg) dissolved in 0.2 mL on the naked backs. The first week after tumor initiation with DMBA, animals receiving DMBA/TPA, DMBA/TPA/Acetone or DMBA/TPA/DADS were further exposed to TPA (4 μg) twice a week for a total 20 weeks. Mice treated with DADS (5 or 20 μmol in 0.2 mL of acetone) were topically applied 30 min before each TPA treatment five times a week until the termination of animal study at 20^th^ week. Tumor size with more than 1 mm diameter was counted every week. Nrf2^−/−^ mice were gifted by Prof. Peng Cao from the Jiangsu Province Academy of Chinese Medicine.

### Histological assessment

After the animals were sacrificed, the skin tissue were isolated and part of the fresh tissues were fixed in 4% paraformaldehyde and sent for hematoxylin and eosin (H&E) staining. Sections were analyzed for the thickness of the epidermis using the ZEN 2011 imaging software on a Zeiss invert microscope under 200-fold magnification.

### Measurement of ROS, GSH and antioxidant enzymes activity

Part of the fresh skin tissues were immediately frozen in liquid nitrogen after excision for further process. Measurement of ROS, GSH and antioxidant enzymes including CAT, SOD, GPx, GR activity was performed using the commercial kits (Beyotime, China).

### RNA isolation and quantitative real-time PCR

Total RNA was extracted from the skin and cells using the TRIzol reagent (Invitrogen). First-strand cDNA was synthesized with 500 ng total RNA using a Hiscript^®^ II QRTSuperMix (Vazyme). Q -PCR was performed using iQ™ RT SYBR^®^ Green supermix and the iQ5 real-time detection system (Bio- Rad Laboratories). The comparative cycle threshold (Ct) method was applied to quantify the expression levels through calculating the 2^(−ΔΔCt)^ method. The primers used for PCR were as follows: GAPDH: 5′-GGTTGTCTCCTGCGACTTCA-3′ (forward) and 5′-TGGTCCAGGGT TTCTTACTCC-3′ (reverse); CAT: 5′-CCCCTATTGCCGTTCGATTCT-3′ (forward) and 5′-TTCAGGTGAGTCTGTGGGTTT-3′ (reverse); SOD: 5′-AACCAGTTGT GTTGTCAGGAC-3′ (forward) and 5′-CCACCATGTTTCTTAGAGTGAGG-3′ (reverse); GR: 5′-GCGTGAATGTTGGATGTGTACC-3′ (forward) and 5′-GTTGC ATAGCCGTGGATAATTTC-3′ (reverse); GPx: 5′-AGTCCACCGTGTATGCCTTC T-3′ (forward) and 5′-GAGACGCGACATTCTCAATGA-3′ (reverse); GCLC: 5′-GGGGTGACGAGGTGGAGTA-3′ (forward) and 5′-GTTGGGGTTTGTCCTCTC CC-3′ (reverse); GCLM: 5′-AGGAGCTTCGGGACTGTATCC-3′ (forward) and 5′-GGGACATGGTGCATTCCAAAA-3′ (reverse); HO-1: 5′-CACGCATATACCCG CTACCT-3′ (forward) and 5′-CCAGAGTGTTCATTCGAGCA-3′ (reverse); NQO1: 5′-TTCTCTGGCCGATTCAGAGT-3′ (forward) and 5′-GGCTGCTTGGAGCAA AATAG-3′ (reverse); Nrf2: 5′-CTCGCTGGAAAAAGAAGTGG-3′ (forward) and 5′-CCGTCCAGGAGTTCAGAGAG-3′ (reverse); HO-1: 5′-CAGGCAGAGAATGCTG AGTTC-3′ (forward) and 5′-GATGTTGAGCAGGAACGCAGT-3′ (reverse); NQO1: 5′-CGCAGACCTTGTGATATTCCAG-3′ (forward) and 5′-CGTTTCTTCCATCCTT CCAGG-3′ (reverse); GAPDH: 5′-TGAAGGTCGGTGTCAACGGATTTGGC-3′ and 5′-CATGTAGGCCATGAGGTCCACCAC-3′.

### Western blot analysis

Whole-cell lysates were prepared with RIPA buffer containing protease and phosphatase inhibitors. Nuclear and cytoplasmic cell extracts were prepared using the NE-PER Nuclear and Cytoplasmic Extraction kit (Thermo). Equal amounts of cell lysates (25 μg) were loaded on SDS-PAGE and transferred onto PVDF membranes. After membranes were blocked, they were incubated with antibody against Nrf2 (1:1000, Abcam), p21 (1:1000, Abcam), Keap1 (1:500, Santa Cruz), LaminB1 (1:1000, Abcam) and GAPDH (1:6000, Bioworld Technology), followed by incubation with Goat anti-Rabbit IgGs -HRP (1:10000, Bioworld Biotechnology). Target proteins were detected by the ECL system (Millipore) and visualized with the ChemiDoc XRS system (Bio-Rad).

### Immunohistochemical (IHC) staining

For IHC analysis of Nrf2 protein, mouse skin was collected and paraformaldehyde fixed, paraffin-embedded sections of skin tissues (4 μm thick) were mounted on slides coated with 2-aminopropyltriethoxysilane, which then were baked, deparaffinized, rinsed with 3% hydrogen peroxide, and incubated with proteinase K (0.5 mg/mL). After that, these sections were washed and then blocked with StartingBlock^TM^ blocking buffers (Pierce, Rockford, IL, USA) for 5 min and subsequently incubated with an anti-Nrf2 (1:100, Abcam) polyclonal antibody for 30 min. Finally, the sections were incubated with Strept Avidin-Biotin Complex (Solarbio) for 30 min at room temperature, followed by detection with a 3,3-diaminobenzidine tetrahydrochoride solution (chromogen) (ZSGB-BIO) and hematoxylin (counterstain). Sections were further mounted with neutral gums. IHC sections were photographed by Mantra 1.01(Perkin Elmer).

### Immunofluorescence staining

After JB6 P+ cells on glass coverslips were treated by indicated agents, they were fixed by pre-cold acetone, then rinsed three times with PBS. The cells were permeabilized in 0.1% Triton X-100 and incubated with 1% BSA/PBS to block nonspecific binding. Subsequently, the cells were immunostained by incubating with Nrf2 antibody (1:100, Abcam) overnight at 4 °C. After being washed with PBS, cells were incubated with Rhodamin-conjugated goat anti-rabbit antibody (1:200, CWBIO, China). Actin filaments were stained using Actin-Tracker (Beyotime, China). And nuclei were counterstained with Hoechst 33258 (Beyotime, China). Fluorescent images were taken and analyzed using the ZEN pro 2012 imaging software on a Zeiss invert microscope under 400-fold magnification.

### Co-Immunoprecipitation assay

For Co-Immunoprecipitation, cell lysates adjusted to 1 mg/mL protein were precleared by antibody p21 (1:20, Abcam) or Keap1 (1:10, Santa Cruz). After gentle rocking at 4 °C overnight, Protein A/G PLUS-Agarose (Santa Cruz) was added to the lysate/antibody mixture, and incubated with gentle agitation at 4 °C for 4 h. Then the immunoprecipitates were collected by centrifugation (12000 rpm, 4 min) and washed three times with cell lysis buffer (NP-40, Beyotime), then boiled for 5 min with the same volume of 2× loading buffer (62.5 mM Tris-HCl, pH 6.8, 2% w/v SDS, 10% glycerol, 50 mM DTT, 0.01% w/v bromophenol blue). Protein interactions were analyzed *via* Immunoblot for Nrf2.

### Transient transfection with p21 siRNA

HEK293T cells plated in 60-mm dishes were transfected with control siRNA or p21 siRNA for 24 h. The transfected cells were then treated with DADS for additional 8 h, followed by Western blot analysis.

### Statistical analysis

Values were expressed as the mean ± SD of at least three independent experiments. One way analysis of variance (ANOVA) was used to compare in groups and p < 0.05 was considered as statistically significant.

## Additional Information

**How to cite this article**: Shan, Y. *et al*. Prophylaxis of Diallyl Disulfide on Skin Carcinogenic Model *via* p21-dependent Nrf2 stabilization. *Sci. Rep.* **6**, 35676; doi: 10.1038/srep35676 (2016).

## Supplementary Material

Supplementary Information
