# Supplementary material for: Prophylaxis of Diallyl Disulfide on Skin Carcinogenic Model via p21-dependent Nrf2 stabilization
Source: Sci Rep. 2016 Oct 19;6:35676. doi: 10.1038/srep35676 (PMC5069634; doi:10.1038/srep35676)
Supplement: Supplementary Information [file srep35676-s1.pdf]

Supplementary Information

Prophylaxis of Diallyl Disulfide on Skin Carcinogenic Model via p21-dependent Nrf2 stabilization

Yunlong Shan, Zhonghong Wei, Li Tao, Siliang Wang, Feng Zhang, Cunsi Shen, Hongyan Wu, Zhaoguo Liu, Pingting Zhu, Aiyun Wang, Wenxing Chen and Yin Lu

Methods

Chemicals and reagents. MK2206 was obtained from Selleck. Antibodies against phospho-Akt, Akt, phospho-ERK, ERK, phospho-P38, P38, phospho-JNK and JNK were procured from Cell Signaling Technology.

Cell line and cell culture. NCI-H1299 (H1299) cell line from ATCC were maintained in RPMI-1640 containing 10% FBS in a humidified 5% CO_2 atmosphere at 37°C.

Anchorage-independent cell growth assay. The pretreated cells (8×10^3/well) were transferred to 1mL of MEM containing 0.35% agar over 3 mL of MEM containing 0.6% agar with 10% FBS in 6-well plates. The cells were maintained with TPA (20 ng/mL) alone or a combination of TPA and various concentrations of DADS in a 5% CO_2 incubator at 37°C for 2 weeks. The cell colonies in soft agar were photographed using a computerized microscope system with Zeiss invert microscope (CarlZeiss) under 40-fold magnification and counted using Image J software.

Supplementary Figure S1

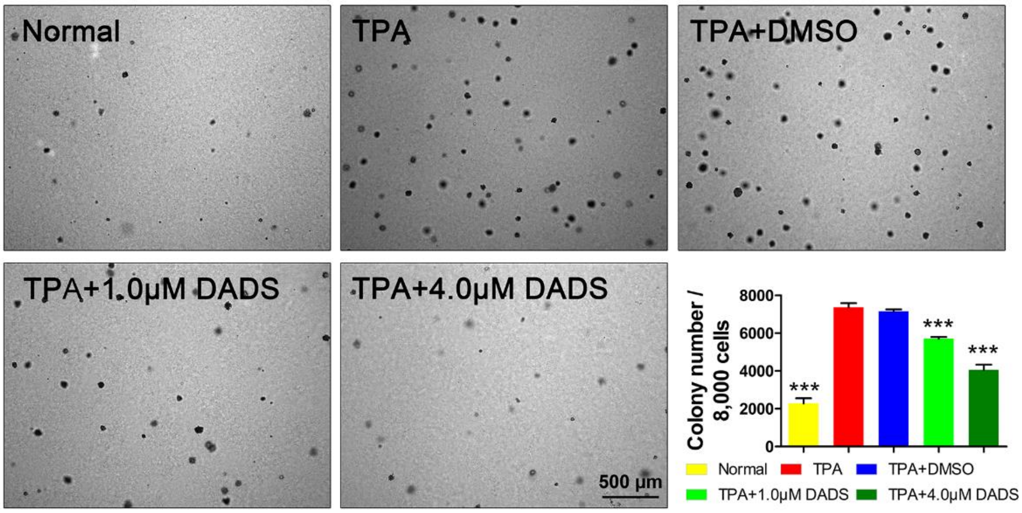

Supplementary Figure S1. The effect of DADS treatment on the TPA-induced anchorage-independent growth of JB6 P+ cells was examined in soft agar. The JB6 P+ cells were pretreated with or without DADS for 5 days. Subsequently, cells incubated with TPA with or without DADS for an additional 14 days for the induction of transformation. The results showed that pretreatment with DADS (1.0 and 4.0 μM) significantly suppressed the TPA induced colony formation of the JB6 P+ cells (n=3). The data are presented as the mean ± SD. ***$p < 0.001$ (versus TPA).

Supplementary Figure S2

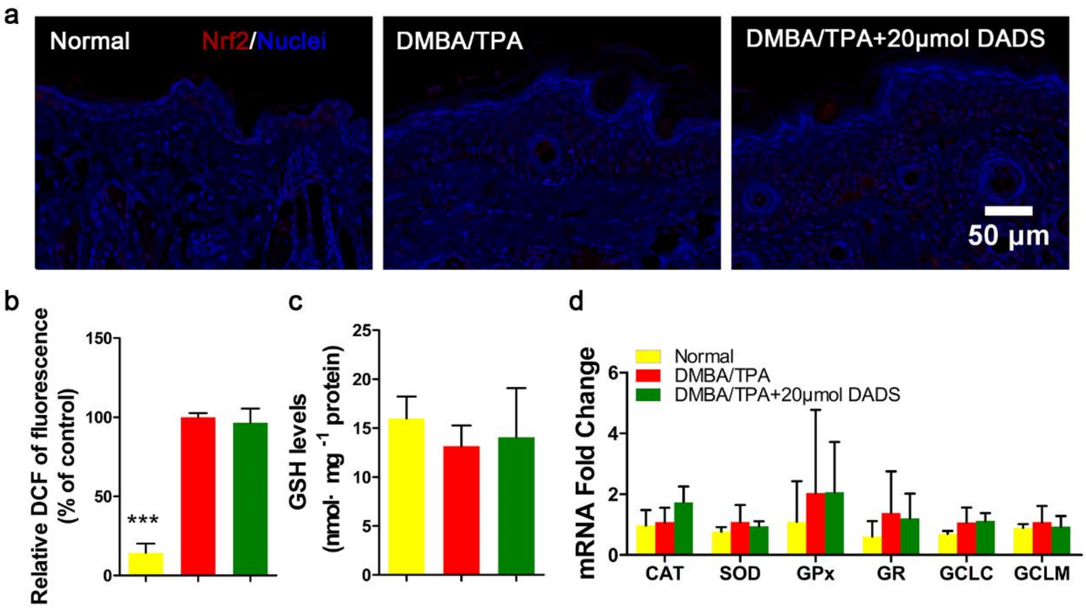

Supplementary Figure S2. DADS failed to induce the Nrf2 nuclear translocation and mRNA levels of Nrf2 target enzymes in Nrf2$^{-/-}$ mice. (a) IHC analysis of Nrf2 expression in mouse epidermis in different treatment groups (blue, nuclei; red, Nrf2). (b) The DCFH-DA staining was used to detect ROS production in the indicated groups (n=3). (c) Effect of DADS on GSH levels in mouse skin from the indicated groups (n=3). (d) Total mRNA was isolated and analyzed to determine the levels of CAT, SOD, GPx, GR ,GCLC and GCLM expression using real-time qPCR after DADS treatment in mouse skin of the indicated groups (n=3). The data are presented as the mean ± SD. (versus DMBA/TPA).

Supplementary Figure S3

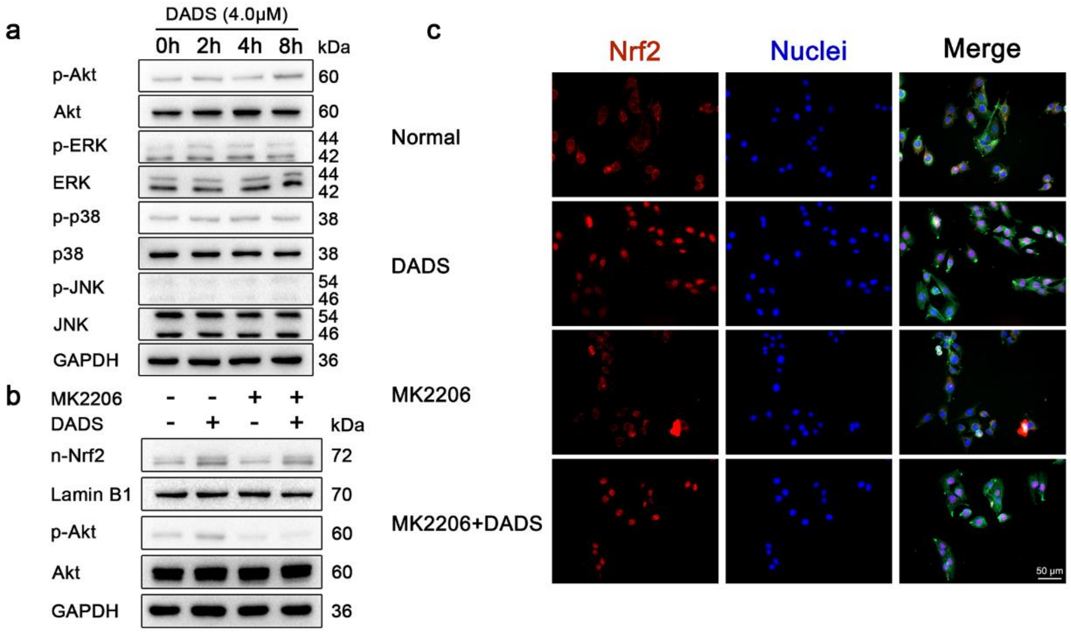

Supplementary Figure S3. DADS failed to induce Nrf2 nuclear translocation through activation of upstream kinases. (a) Cells were incubated with DADS for the indicated time, and the expression of both phosphorylated and total forms of Akt, ERK1/2, p38 and JNK1/2 were measured by Western blot analysis. (b) Cells were pre-treated with 40 nM of MK2206, an inhibitor of p-Akt, followed by DADS treatment for 8 h. The nuclear protein extract was subjected to immunoblot analysis for the detection of Nrf2 expression. (c) Treatment was similar to (b). Immunofluorescence analysis of Nrf2 was carried out as described in Materials and Methods. GAPDH and Lamin B1 were used as the loading control.

Supplementary Figure S4

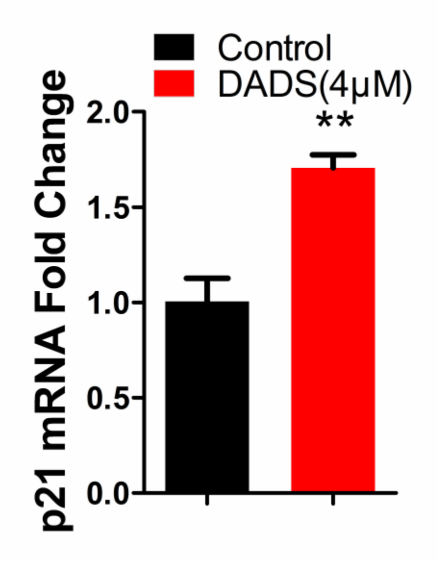

Supplementary Figure S4. DADS induced the mRNA levels of p21 in cells. Total mRNA was isolated and analyzed to determine the levels of p21 expression using real-time qPCR after DADS treatment (n=3). The data are presented as the mean ± SD. (versus Control).

Supplementary Figure S5

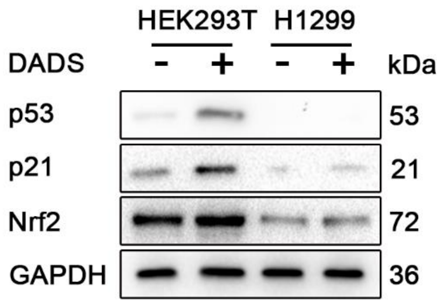

Supplementary Figure S5. DADS failed to induce Nrf2 in H1299 cells. HEK293T and H1299 cells were incubated with DADS for 8 h to determine the levels of p53, p21 and Nrf2 by Western blot analysis. GAPDH was used as the loading control.

Supplementary Figure S6

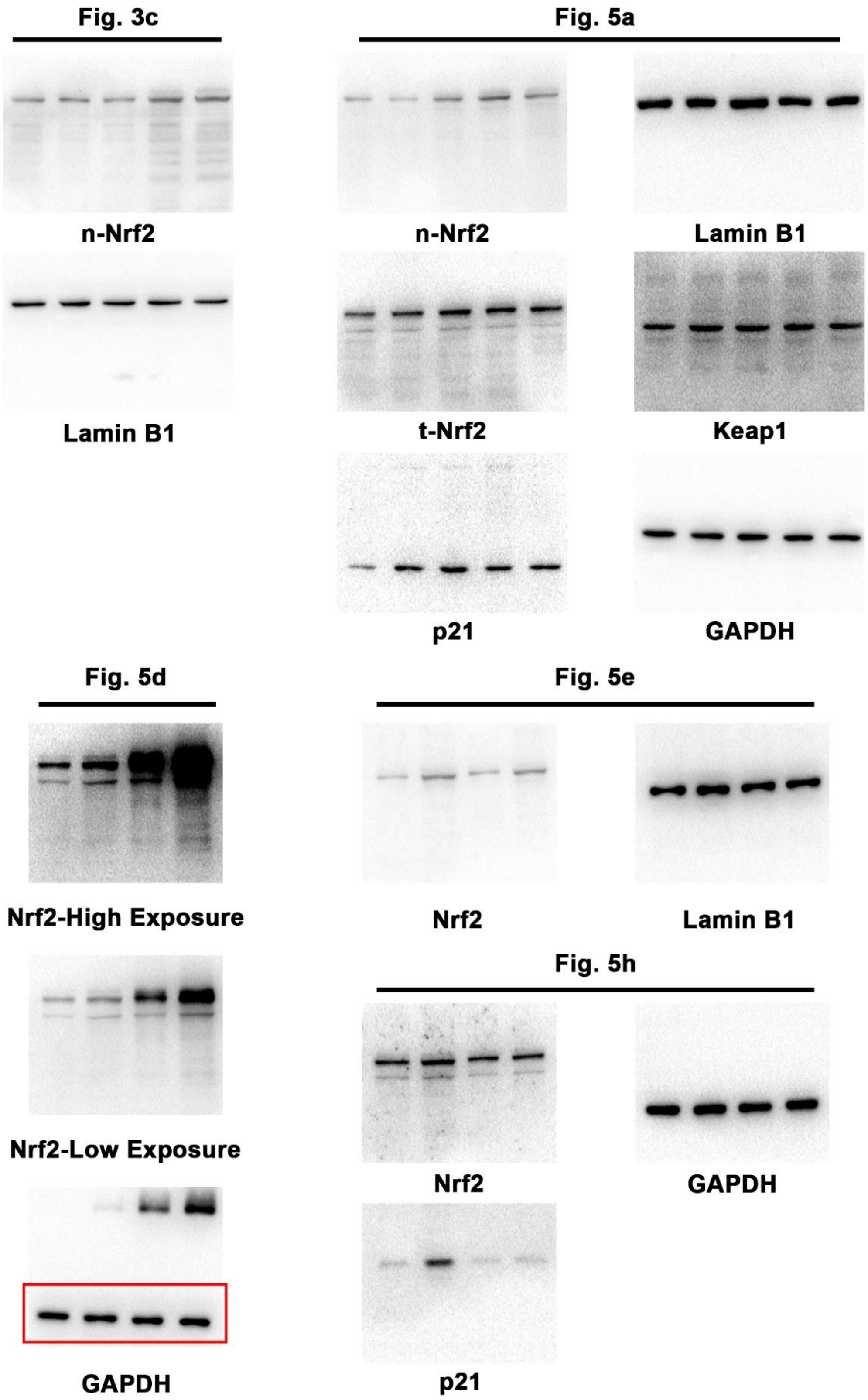

Supplementary Figure 6. Full-length blots in this study.

Supplementary Figure S7

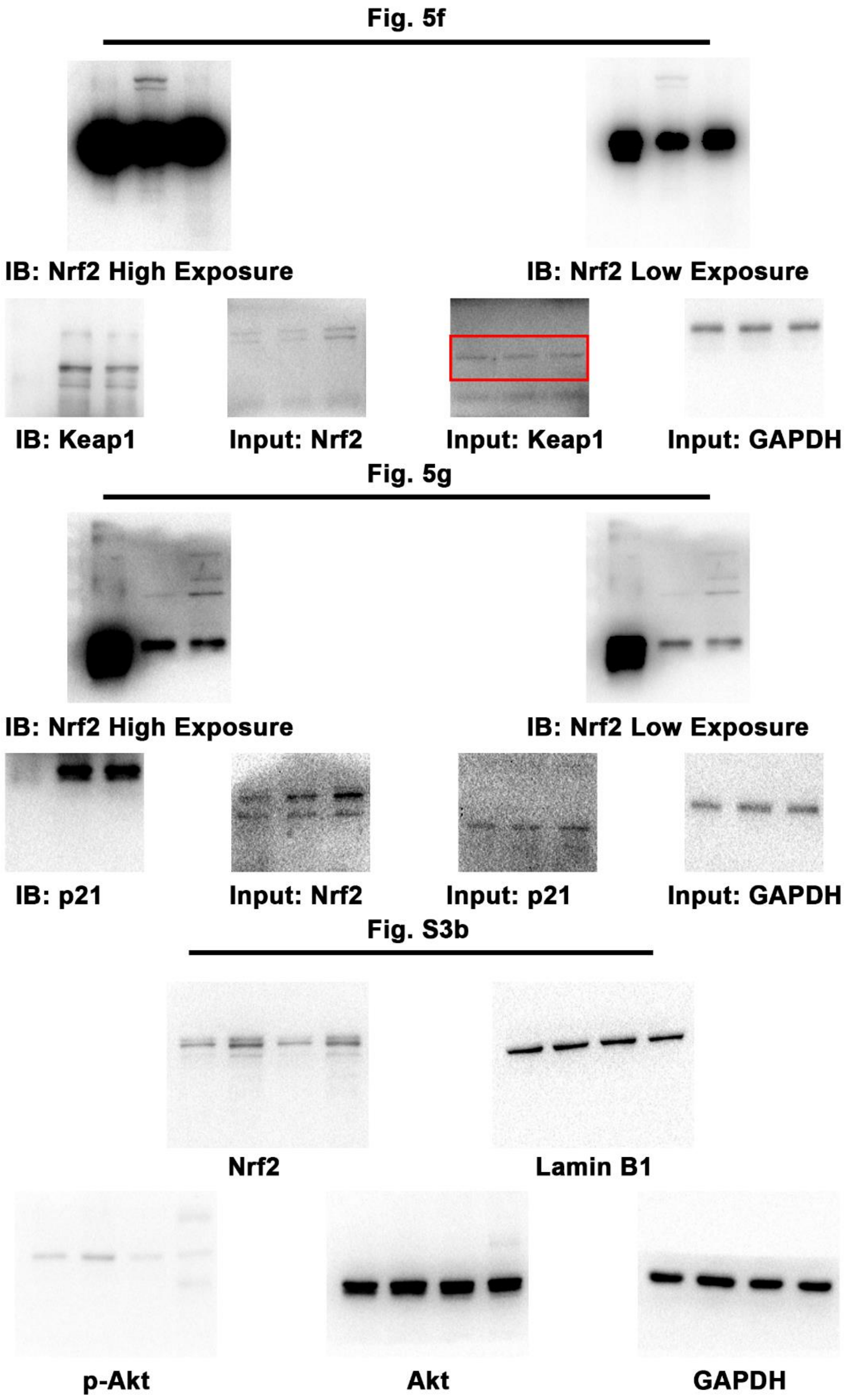

Supplementary Figure 7. Full-length blots in this study.

Supplementary Figure S8

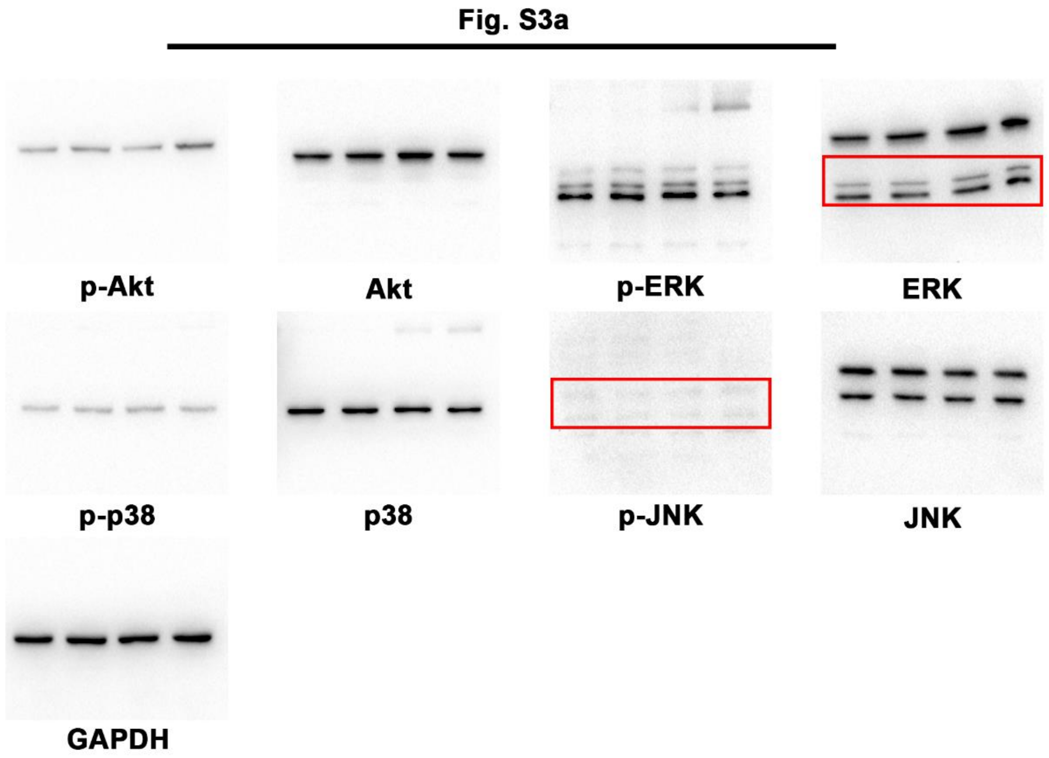

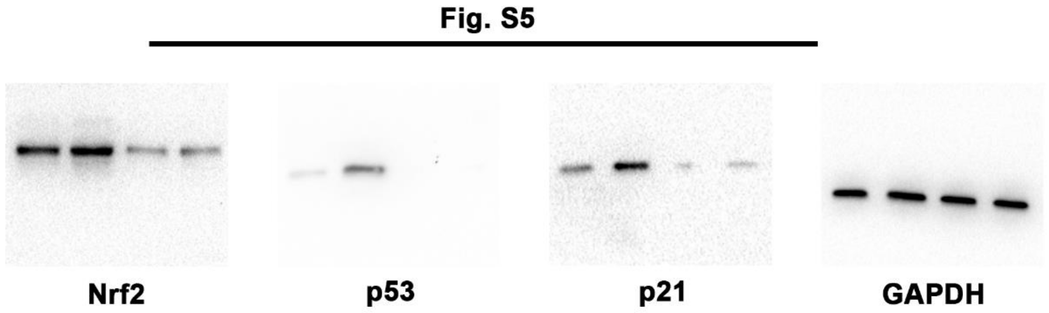

Supplementary Figure 8. Full-length blots in this study.
